# Homœopathy and Regular Medicine

**Published:** 1871-08

**Authors:** H. R. Hopkins

**Affiliations:** Buffalo


### Homœopathy and Regular Medicine,

*To the Editor of the Buffalo Medical and Surgical Journal*:

It will be to the advantage of the regular medical profession to go carefully over their treatment of the class of physicians who have seen fit to denominate themselves homœopathic, and to observe the effect such treatment has had upon the profession itself, upon the public and upon homœopathy.

That the accumulated experience of faithful observers, who, for the last four thousand years have given their lives to the study and treatment of disease, is, we believe, of almost invaluable importance to one who wishes to become a physician, and certainly is of infinite importance when compared with a hypothetical dogma, and yet, with all the machinery of our hospitals and dispensaries, the control of every medical appointment in the gift of governments or corporations, with our medical schools perfectly equipped with professors for every separate department of medicine, and an entire monopoly of the advantages of clinical observations, with all these advantages and precedents, what headway have we made in convincing the public and individuals of our superior ability to manage diseases, or of our peculiar fitness for becoming the sanitary officers of households or communities?

The line of treatment which the regular profession saw fit to adopt in the earliest days of homœopathy, and which they are still following, is generally bigoted, and universally intolerant opposition. What is the effect of this opposition? It is to arouse in the public mind that generous American sentiment which ever asserts itself to see fair play between a big boy and a little one. There is scarcely an instance in which the regular profession, with all its accumulated prestige, has arrayed itself against homœopathy, where the weaker party have not prevailed. And to-day, in the sight of the law, and in the confidence of the people, homœopathy is the peer of regular medicine.

It becomes us to go over this case, and, if possible, discover why, we so strong in numbers, and in all the facilities and appliances for illustrating and enforcing our tenets, are so repeatedly beaten? Why is it that individuals and corporations are becoming convinced that their interests require them to employ homœopathic in preference to regular physicians? For myself, in spite of the logic of events, I still believe, and my belief is founded upon a thorough investigation of the principles of homœopathy, and observations upon the practice of many of its most distinguished disciples, that in no way can a man so efficiently equip himself for the responsibility of the management of disease, and the custody of health as in the study of regular medicine.

If we take it for granted that the past experience and observations of physicians are of service to physicians at present, and I do not think we will be charged with assumption, for considering this an axiom; then why is it that a sect which disregards all traditions of medicine, and found their system upon a dogma which contradicts all that we have held as truth, why is it that they are flourishing and we are going to the wall?

The answer to this question presents itself to my mind under two heads, which may be formularized as follows: Homœopathy lives upon the disgrace brought upon the profession of medicine by the low standard of medical education, and flourish upon the intolerant opposition it has received at the hands of regular physicians.

It is with the second, the lesser of the two evils I propose to deal at this time.

The treatment of homœopathy by the regular profession in past years is so well known as to require no mention, therefore let us turn our attention to the present, and by reading its signs in the light of the past, endeavor to do something for our future.

The position of the regular profession in regard to homœopathy may be expressed in few words. We are not aware of their existence. They have no professional rights which we are bound to respect, and when forced by some laymen to speak upon the subject, or give an opinion upon homœopathy, the opinion is that it is a "a humbug." This line of treatment was bad enough when homœopathy was young, but now when we stand on equal footing before the law, and nearly equal before the public, it is suicidal.

It may be well to explain what I mean by equal rights before the law. All the rights which members of the regular profession of this State enjoy are granted them by Acts of Legislature, the first of which was passed April 10th, 1813, this and the Act of 1827, contain the "Regulations concerning the Practice of Physic and Surgery in this State." They provide for the establishment of County Medical Societies, "the only organization existing under law for the purpose of diffusing true science and the knowledge of the healing art," and otherwise point out and fix the duties, responsibilities and immunities of physicians and surgeons.

On April 13th, 1857, the Legislature of this State admitted the homœopathic profession to all the rights and privileges enjoyed by members of the regular profession under the above mentioned Acts. This provided for the present, and in the Acts incorporating their colleges, exactly the same powers are granted to them as had been granted to our medical schools, which provides for the future. I doubt not there are members of our profession who have hitherto failed to realize the change wrought in the homœopathic profession by the Act of 1857. As before stated, that Act admitted the homœopathic profession to all the rights and privileges as physicians and surgeons under the Acts of 1813 and 1827, and all Acts amendatory thereof, thus they became "legally authorized practising physicians and surgeons," and as such, are entitled to membership of our County Medical Societies. This right is positive, and no County Society has the power to adopt a by-law which will keep them out if they should make application for admission. The right of legally authorized physicians to membership of County Medical Societies has been most definitely settled by our courts, and the proceedings to obtain such rights are well understood by many of our members.

In view of these facts, what should the regular profession do in the matter. Shall we continue to call ourselves "the profession," and neither by public act or private word allow that there is any other? Shall we continue a line of treatment condemned by law and by experience, treatment which only makes homœopathy notorious and ourselves disgraceful; or shall we submit gracefully to the laws of the State, and public opinion, and proffer to the homœopathic profession those amenities which should exist between professional equals? Invite them to their rights in our County Medical Societies, when called by their patrons, attend with them in consultation; when wished by our patients ask them to attend in consultation with us. If they have any superior knowledge in the management of disease or the protection of health, our duty to our patrons requires us to avail ourselves of that knowledge. If we possess the greater professional ability, they and their patrons will find it out. If we hold back from this, we may reasonably be charged with having little confidence in our doctrines. If we go into it; I rest my faith upon "the survival of the fittest."

Buffalo, August 1871. H. R. Hopkins, M. D.

